# Simultaneous improvement of breast muscle yield and meat quality in Langshan chickens

**DOI:** 10.1016/j.psj.2026.106552

**Published:** 2026-01-30

**Authors:** Junjie Chen, Xiuze Zhang, Lin Zhang, Tong Xing, Feng Gao, Liang Zhao

**Affiliations:** a College of Animal Science and Technology, State Key Laboratory of Meat Quality Control and Cultured Meat Development, Key Laboratory of Animal Origin Food Production and Safety Guarantee of Jiangsu Province, Jiangsu Collaborative Innovation Center of Meat Production and Processing, Quality, and Safety Control, Nanjing Agricultural University, Nanjing 210095, PR China; b College of Food Science and Technology, Nanjing Agricultural University, Nanjing 210095, PR China

**Keywords:** Chicken, Breast muscle yield, Myogenic capacity, Meat quality, Unsaturated fatty acids

## Abstract

Compared to white-featured broilers, the native Chinese Langshan chickens possess superior meat quality but have a lower growth rate and breast muscle yield. Increasing the breast muscle yield of these breeds while maintaining their high meat quality would greatly enhance their commercial value. Although elevated muscle growth often impairs meat quality, this relationship remains unclear in Langshan chickens. In the present study, 15-week-old Langshan chickens were divided into high (HPB, 14.15 %) and low (LPB, 9.85 %) percentages of breast muscle yield groups after slaughter. Differences in meat quality, myogenic capacity, protein deposition capacity, and metabolic profiles were investigated. Interestingly, the results demonstrated that, compared to the LPB group, the HPB group exhibited improved meat quality with reduced shear force (19.05 N vs 22.63 N) and drip loss (2.50% vs 3.07 %). The HPB group showed a decreased average muscle fiber diameter and an increased muscle fiber density, suggesting increased numbers of muscle fibers. Moreover, immunostaining revealed a higher number of PAX7^+^, MYOD1^+^, and PAX7^+^/MYOD1^+^ satellite cells in the HPB group, accompanied by elevated gene expression of *MYF5, MYOD1*, and *MRF4*. Consistently, protein deposition capacity was enhanced as genes and proteins related to protein synthesis were upregulated while genes and proteins related to degradation were downregulated. Untargeted metabolomic profiling identified 253 differential metabolites. Enrichment analysis of up-regulated differential metabolites in the HPB group identified pathways such as unsaturated fatty acid biosynthesis and arachidonic acid metabolism, suggesting a potential function of unsaturated fatty acids in myogenic regulation. We also validated the positive effects of a key differential metabolite, docosapentaenoic acid (DPA), on the myogenic differentiation of SCs by in vitro cell culture experiments. Collectively, these findings provide novel insights that enhancing breast muscle yield did not impair meat quality but instead improved meat tenderness and water-holding capacity, an effect that may be attributable to increased numbers but not sizes of muscle fibers. Therefore, this work establishes a theoretical basis for simultaneously improving breast muscle yield and meat quality in chickens.

## Introduction

The breast muscle is the main cut in broiler products and significantly impacts the economic benefits of commercial operations. Consequently, the broiler industry tends to select breeds with fast growth rates and high breast muscle yield (Maharjan et al., 2021; Velleman et al., 2015). However, pursuing higher muscle growth rate and breast muscle yield often compromises intramuscular fat content and overall meat quality. Customers in Asian countries prefer local chicken breeds with superior meat quality and rich flavors. Compared to the white-feathered commercial broilers, the local breeds in Asian normally have better meat quality but a slower growth rate and lower breast muscle yield. Therefore, strategies for the simultaneous improvement of muscle growth and meat quality in local chicken breeds have become a major focus for the industry.

To achieve above goals, we must better understand the composition of skeletal muscles in chickens and the regulatory mechanisms for their growth. Satellite cells (SCs) are muscle stem cells located between the basement membrane and sarcolemma of muscle fibers, playing a key role in the growth, repair, and regeneration of skeletal muscle. Under the stimulation of muscle injury or growth signals, SCs are activated from a quiescent state, undergo proliferation and differentiation, and finally fuse with existing muscle fibers or with each other to form new muscle fibers, thereby promoting muscle hypertrophy and repair (Sousa-Victor et al., 2022; Kuang et al., 2008). The myogenic capacity of SCs is regulated by a coordinated network of myogenic regulatory factors, including *PAX7, MYF5, MYOD1, MYOG*, and *MRF4* (Relaix et al., 2012; Brack et al., 2012).

The hypertrophy of muscle fibers in chickens is primarily driven by the continuous incorporation of new nuclei from SCs during postnatal growth, which enhances protein synthesis. The regulation of protein synthesis in skeletal muscle is strictly controlled by various factors, including growth factors, hormones, nutrients, and exercise (Miyazaki et al., 2009; Suryawan et al., 2011). Among these, the mammalian target of rapamycin (mTOR) signaling pathway has become a core regulator of protein synthesis in response to nutrient supply and growth factor stimulation (Laplante et al., 2012). Activation of the mTOR pathway leads to the phosphorylation and activation of downstream effectors, such as p70 ribosomal S6 kinase (p70S6K) and eukaryotic translation initiation factor 4E-binding protein 1 (4E-BP1), thereby promoting the initiation and elongation of protein synthesis (Nicklin et al., 2009; Laplante et al., 2012). In addition, the biological process of protein degradation affects the net protein contents in the skeletal muscle. Protein degradation is mainly mediated by the ubiquitin-proteasome system (UPS) and the autophagy-lysosome pathway. The activity of these pathways must be maintained within a certain safe range to avoid muscle atrophy (Gumucio et al., 2013).

Accumulating evidence indicates that metabolites—small molecules produced by cellular metabolism—also exert a profound impact on the growth and development of skeletal muscle. Metabolites can act as signaling molecules, energy sources, or substrates for biosynthetic pathways, thereby regulating various cellular processes such as cell proliferation, differentiation, and metabolism. For example, recent studies have shown that certain metabolites, such as lactic acid, can regulate SC function and muscle regeneration by activating specific signaling pathways (Bartoloni et al., 2024). Consequently, discovering endogenous metabolites that regulate muscle growth is crucial for developing nutritional strategies to enhance breast muscle yield.

It is well documented that intensive selection for rapid growth and high breast muscle yield in white-feature broilers is associated with an increased risk of emerging myopathies including white striping and wooden breast (Huang X et al., 2018). In addition, abnormal post-mortem pH was associated with higher breast muscle yield, which impairs meat quality. However, the severity of these quality issues tends to increase markedly only in birds with very high breast yields and large fiber cross-sectional areas, which place greater metabolic and structural stress on the pectoralis major (Huang X et al., 2018). The present study focused on Langshan chickens, a native breed from China that has not been extensively selected for commercial production traits. Compared to white-feathered broilers, Langshan chickens have a significantly lower breast muscle yield. Increasing the breast muscle yield within a certain range in Langshan chickens may not negatively impact meat quality, as often occurs in white-feathered broilers. In addition, increasing the breast muscle yield of Langshan chickens while maintaining their high meat quality would greatly enhance their commercial values. Therefore, the objective of this study is to evaluate the influence of increased breast muscle yield on meat quality traits and muscle fiber characteristics and explore potential causing mechanisms by examining the myogenic capacity, protein deposition capacity, and metabolite compositions. Results from this study will provide valuable information for the production of high-quality local chicken breeds with increased breast muscle yield and superior meat quality.

## Materials and methods

### Animals and sample collection

All poultry slaughtering and experimental procedures in this study were approved by the Animal Care and Use Committee of Nanjing Agricultural University (Approval No.: NJAU.No20230516074). Langshan chickens were provided by Nantong Rudong Langshan Breeding Farm (Nantong, Jiangsu, China). Commercial feed was used in the experiment (Table 1). Seventy 15-week-old male Langshan chickens with uniform body weight were selected. All birds were obtained from a single Langshan population maintained at one breeding farm and belonged to the same generation. After 12 h of fasting (with free access to water), blood samples were collected from the wing vein using vacuum blood collection tubes, centrifuged at 3000 rpm for 10 min at 4°C. The supernatant was aspirated and stored in 1.5 mL centrifuge tubes at −20°C. Subsequently, the chickens were stunned with carbon dioxide, sacrificed by exsanguination via the jugular vein, and the left and right pectoral muscles were collected. For the left pectoral muscle, molecular samples were taken and quickly stored in liquid nitrogen for subsequent experiments, while fixed samples were preserved in 4 % paraformaldehyde (G1101, Servicebio, Wuhan, China). The right pectoral muscle was stored at 4°C for subsequent meat quality determination. Based on the slaughter performance measurements, the 70 chickens were ranked according to breast muscle percentage, and the 7 birds with the lowest values were assigned to the LPB group, whereas the 7 birds with the highest values were assigned to the HPB group.

**Table 1:** Feed ingredients and Nutrient levels of basal diet.

Ingredient (%)	1-21 days of age	22-105 days of age
Corn	60.00	63.54
Soybean meal	32.85	27.49
Corn gluten meal (56 %, CP)	1.92	3.00
Soybean oil	1.46	2.03
Dicalcium phosphate	1.40	1.72
Limestone	1.26	1.24
Salt	0.30	0.32
L-Lysine	0.20	0.13
DL-Methionine	0.20	0.11
Choline chloride (50 %)	0.19	0.20
Vitamin-mineral premix^1^	0.22	0.22
**Nutrient level**^2^		
Metabolizable energy (MC/Kg)	12.01	12.55
Crude protein (%)	20.00	19.00
Calcium (%)	1.00	0.90
Total phosphorus (%)	0.69	0.63
Available phosphorus (%)	0.45	0.40
Lysine (%)	1.05	1.00
Methionine (%)	0.48	0.43
Threonine (%)	0.74	0.76
Tryptophan (%)	0.22	0.23

1 Premix provided per kilogram of feed: VA 9000 IU, VD_3_ 2700 IU, VE 20 mg, VB_1_ 3.0 mg, VK_3_ 2.4 mg,VB_2_ 6.4 mg,VB_6_ 2.8 mg, VB_12_ 0.01 mg, D-pantothenic acid 11 mg, folic acid 0.70 mg, biotin 0.08 mg, niacin 40 mg, choline 460 mg, Mn 60 mg, Fe 40 mg, Cu 10 mg, Zn 55 mg, I 1.6 mg, Se 0.35 mg.

2 Calculated values.

### Determination of slaughter performance

The live weight was measured before sacrifice. After slaughter, the determination methods of dressing percentage, and percentages of half-eviscerated yield, eviscerated yield, breast muscle yield, leg muscle yield, and abdominal fat percentage yield referred to the Terminology and Measurement Statistical Methods for Poultry Production Performance of China (NY/T823-2020).

### Determination of meat quality

As previously described (Wang et al., 2024), the pH value was measured by a portable pH meter (FiveGoTM, MettlerToledo, Switzerland) at 45 min and 24 h post-slaughter. Meat color of lightness (L*), redness (a*), and yellowness (b*) was detected at 24 h post-slaughter by a colorimeter (CR-400, KonicaMinolta, Japan). Drip loss, cooking loss, and shear force were measured at 24 h post-slaughter following our previous reported method (Zhao et al., 2018, 2016).

### Histological analysis

The measurement of muscle fiber histological morphology was based on the method described in a previous study (Patael et al., 2019). Briefly, cross-sectional sections of pectoral muscle tissue were stained with hematoxylin and eosin (G1076, Servicebio, Wuhan, China) and examined under a microscope (NIKON ECLIPSE E100, Nikon, Tokyo, Japan). Image Pro Plus software (Media Cybernetics, Bethesda, MD, USA) was used to analyze the muscle fiber diameter and density. Sections were observed under 10 × 10 and 10 × 20 magnification fields, and 3 random fields were selected for each magnification. Muscle fiber number = Left pectoral muscle weight / Average muscle fiber cross-sectional area" (with exact muscle weight measured to 0.01 g).

### Immunofluorescence staining of satellite cells (SCs)

The immunofluorescence procedure for SC identification was performed essentially as described by Feng et al. (2018). Tissue sections were dewaxed with xylene and rehydrated through graded ethanol, and were immersed in 10 mM sodium citrate buffer (pH 6.0; 2.94 g/L trisodium citrate dihydrate and 0.42 g/L citric acid monohydrate in distilled water) and heated in a microwave oven. The buffer was brought to a gentle boil at medium power (approximately 700 W) for 10 min and then maintained at sub-boiling temperature at medium–low power (approximately 400 W) for an additional 10 min, followed by cooling in the same buffer at room temperature for 20 min before PBS washing. After PBS washing, tissues were circled with a hydrophobic pen and blocked with 3 % BSA for 30 min at room temperature. Mixed primary antibodies (DSHB, IA) were applied and incubated overnight at 4°C, followed by PBS washes and incubation with corresponding secondary antibodies (Beyotime Biotechnology, Shanghai, China) for 50 min in the dark. Nuclei were stained with DAPI for 10 min, and autofluorescence was quenched. Finally, sections were mounted with anti-fade medium and observed under a fluorescence inverted microscope.

The primary antibodies used in the immunofluorescence experiment were as follows: mouse anti-PAX7 monoclonal antibody (DSHB, IA), rabbit anti-MYOD1 polyclonal antibody (Affinity Biosciences, Melbourne, Australia). The secondary antibodies used in the immunofluorescence test are as follows: CY3-labeled goat anti-mouse IgG (Beyotime Biotechnology, Shanghai, China), Alexa Fluor® 488-conjugated goat anti-rabbit IgG (Beyotime Biotechnology). Images were acquired by using a Nikon Eclipse Ti-U fluorescence inverted microscope (Nikon Instruments, InC., Mellville, NY) at 200 × magnification. For each sample, 5 non-overlapping fields of view were randomly selected, excluding areas with tissue tears and incomplete fiber cross-sections; counts were independently performed by two observers unaware of the group information, and the average was taken. The numbers of DAPI^+^, PAX7^+^, MYOD1^+^, PAX7^+^/MYOD1^-^, and PAX7^+^/MYOD1^+^SCs were quantified through ImageJ.

### Determination of serum hormone levels

After collecting blood from the wing vein, the samples were centrifuged at 3000 rpm for 10 min at 4°C. The supernatant was aspirated and stored in 1.5 mL centrifuge tubes at −20°C for subsequent analysis. Commercial ELISA kits (AiFANG Biological, Hunan, China) were used, including triiodothyronine (T3) ELISA kit, thyroxine (T4) ELISA kit, and insulin-like growth factor-1 (IGF-1) ELISA kit. The specific operation steps were performed according to the instructions provided by the supplier.

### RT-qPCR

Total RNA was extracted from the molecular samples of pectoral muscle using RNAiso Plus reagent (9108, Takara, China), and the purity and concentration of total RNA were measured using a spectrophotometer (NanoDrop 2000; Thermo Fisher Scientific, USA). A commercial kit (Jiangsu Cowin Biotech Co., Ltd, Jiangsu, China) was used for reverse transcription of the samples. Quantitative real-time PCR (qPCR) was performed using an Applied Biosystems StepOnePlus Real-Time PCR System, with SuperStar Universal SYBR Master Mix (Jiangsu Cowin Biotech Co., Ltd, Jiangsu, China) and gene-specific primers (Table 2). The relative gene expression levels were calculated using the 2^-ΔΔCT^ method. All RT-qPCR reactions were performed with three technical replicates for each biological sample. The mean Ct value of the technical replicates was used for subsequent analysis. Gene expression levels were normalized to GAPDH as the internal reference gene.

**Table 2:** Primer sequence of target genes and housekeeping gene.

Genes	Gen Bank numbers	Primer sequences (5′→3′)	Product lengths
*PAX7*	NM_205061.5	F:AGGGAGAACCCTGGGATGTT R:GCACACGGCTAATCGAACTC	106
*MYF5*	NM_001030363.2	F:CCAGAGACTCCCCAAAGTGG R:CCACCTGTTCCCTCAAGAGC	83
*MYOD1*	NM_204214.3	F:CCCATGGAAATGACGGAGGG R:TGAAGCACGGGTCGTCATAG	73
*MYOG*	NM_204184.2	F:TCCCGGAGCAGAGGTTTTAC R:CTCGGGACGCTCAGGAAATG	89
*MRF4*	NM_001030746.3	F:CTGCAAGAGAAAGTCGGCCC R:GTCCGCCTTTTCAGAGCCT	108
*mTOR*	XM_040689168.2	F: GGTCTTTGGGGATCTGGTGA R: CCCTTTTGCCTCCTATGCCA	268
*IGF-1R*	NM_205032.3	F:ACCAGGTCTTTCTCAGCACG R:GGCAAACTGGGCTTCACTTG	123
*4EBP1*	XM_424384.8	F: CGGGCGGAACCAGGATTATT R:GTCCGGAAGGTCAGAAGGTG	92
*IRS-1*	XM_040679294.2	F: GAGGACAGCTATGGCGAGGT R: TTAGTCAGGCACAGGCGGTA	125
*S6K1*	XM_046902412.1	F: TGGACCATGGAGGAGTTGG R: AGCACTCCGGTCGGATCTT	111
*MAFBX*	NM_001039309	F: CCAACAACCCAGAGACCTGT R: GGAGCTTCACACGAACATGA	180
*MURF1*	XM_204639	F:ACCCCCAACCCCATGATCCAG R: TACACTGCTGTGGCCCCCAT	160
*TRα*	XM_014192391.2	F: GCTGCTGCATCATCGACAAG R:GCTACCCGCTTAGAGTCGTC	115
*TRβ*	NM_001252221.2	CCGAAAACACCACGTGACAC R:GCATGGCAGGCTCCTATCAT	81
*PLIN1*	NM_001127439.1	F:GCCAAGGAGAACGTGCT R:TCACTCCCTGCTCATAGACC	142
*FABP4*	NM_204290.1	F:GGGGTTTGCTACCAGGAAGATG R:CATTCCACCAGCAGGTTCCC	276
*LPL*	NM_205282.1	F: AGGAGAAGAGGCAGCAATA R:AAAGCCAGCAGCAGATAAG	222
*LDLR*	NM-204452	F:CGGAAGAAGGAGAAGGGG R:CCTCATCAGAGCCGTCAGC	258
*CD36*	NM_001030731	F:TACCAGACCAGTAAGACCGTGAAGG R:AATGTCTAGGACTCCAGCCAGTGT	156
*PCNA*	NM_204170.2	F:AACACTCAGAGCAGAAGAC R: GCACAGGAGATGACAACA	225
*CCND1*	NM_205381.1	F:CTCCTATCAATGCCTCACA R: TCTGCTTCGTCCTCTACA	165
*CDK2*	NM_001199857.1	F:CCAGAACCTCCTCATCAAC R: CAGATGTCCACAGCAGTC	171
*MYHC*	XM_015295778.2	F:GAAGGAGACCTCAACGAGATGG R: ATTCAGGTGTCCCAAGTCATCC	138
*MEF2C*	XM_004949410.3	F:TAGGTCACAGCCCTGAGTCT R: ATGGGCCAGTGGCAAAAGAT	203
*GAPDH*	NM_204305.2	F:CTTTGGCATTGTGGAGGGTC R:ATGACTTTCCCCACAGCCTT	162

### Western blotting

Frozen muscle tissue was lysed, homogenized, and centrifuged at 12,000 × *g* for 10 min at 4°C. The supernatant's protein concentration was measured by BCA, normalized to 4 μg/μL, and mixed with 5 × loading buffer (4:1 ratio). The mixture was heated at 100°C for 5 min. Equal volumes (6 μL) were loaded onto a 10 % SDS-PAGE gel (or 6 % for >200 kDa proteins), then transferred to PVDF membranes. Membranes were blocked with 5 % BSA for 2 h, then incubated overnight at 4°C with primary antibodies: β-Tubulin (A12289, rabbit monoclonal antibody from Abclonal), mTOR (A11345, rabbit polyclonal antibody from Abclonal), Phospho-mTOR-S (AP1413, rabbit monoclonal antibody from Abclonal), and Murf1 (A3101, rabbit polyclonal antibody from Abclonal). After washing, membranes were incubated with HRP-conjugated secondary antibody for 2 h. Chemiluminescence was detected with BeyoECL Plus, and band intensities were analyzed using Image Pro Plus, with β-Tubulin as the internal control.

### Untargeted metabolomics analysis

Approximately 100 mg of pectoral muscle was extracted with 400 μL acetonitrile:methanol (1:1) using low-temperature ultrasonication (5°C, 30 min). After centrifugation (15,000 × *g*, 20 min, 4°C), the supernatant was diluted to 53 % methanol, centrifuged again, and analyzed by LC-MS/MS (Q Exactive™ HF, Thermo Fisher Scientific, USA).Chromatographic separation was performed on a Hypersil Gold C18 column (40°C, 0.2 mL/min) with mobile phases of 0.1 % formic acid (A) and methanol (B). Both positive and negative ion modes were applied under standard ESI conditions. QC samples were prepared by pooling equal aliquots from all samples and analyzed every 10 injections. Data were processed using metaX for PCA, PLS-DA, and differential metabolite screening based on VIP > 1, *P* < 0.05, and fold change criteria.

Differential metabolite analysis: The metabolomics data processing software metaX was used for data transformation. Principal component analysis (PCA) and partial least squares discriminant analysis (PLS-DA) were performed to obtain the variable importance in projection (VIP) value of each metabolite. For univariate analysis, the t-test was used to calculate the statistical significance (p-value) of each metabolite between the two groups, and the fold change (FC) of metabolites between the two groups was calculated. Potential differential metabolites were selected based on the following criteria: |log₂ fold change (FC)| ≥ 1, VIP value from OPLS-DA model ≥ 1, and *P*-value ≤ 0.05.

### In vitro culture of chicken satellite cells

Skeletal muscle SCs were isolated from the pectoralis major muscle of 12-day-old Langshan chicken embryos as previously described, with minor modifications (Harding et al., 2016). Briefly, pectoral muscles were excised under sterile conditions, minced, and enzymatically digested with collagenase type II and trypsin. The cell suspension was filtered through a 70-μm cell strainer and pre-plated to reduce fibroblast contamination. SCs were cultured in high-glucose DMEM (Gibco, Grand Island, NY, USA) supplemented with 20 % fetal bovine serum, 10 % horse serum, and penicillin/streptomycin. A gradient concentration of 0, 0.5, 5, or 50 μM docosapentaenoic acid (DPA) were selected for treatment. To evaluate the effects of DPA on proliferation, SCs were seeded in growth medium and treated with different concentrations of DPA for 24 h. In a separate experiment, to assess the effects of DPA on the differentiation of SCs, cells were cultured until reaching 80–90 % confluence, after which the growth medium was replaced with differentiation-induction medium consisting of DMEM supplemented with 2 % horse serum. Cells were then treated with DPA at the indicated concentrations for 48 h. Dimethyl sulfoxide (DMSO)–treated cells served as vehicle controls in all experiments, and the final concentration of DMSO did not exceed 0.1 %.

### Statistical analysis

Comparisons between two groups were performed using independent samples t-test of SPSS (version 25.0, SPSS Inc., Chicago, USA). Each bird (*n* = 7 per group) was considered one biological replicate for slaughter performance, meat quality, histological measurements, serum hormones, gene expression and metabolomic analyses. For in vitro cell culture experiment, three independent studies were conducted (*n* = 3). RT-qPCR reactions were run in technical triplicate and the mean Ct value was used for analysis. Data were expressed as mean ± SEM. GraphPad (Prism 8.3.0, Insightful Science, California, USA) were used for visualization. Statistical significance was determined at a threshold of *P* < 0.05.

## Results

### Comparison of slaughter performance

Based on slaughter performance data of 70 Langshan chickens with comparable body weights, two groups (7 chickens for each group) with high (HPB) or low (LPB) percentage of breast muscle yield were selected in this study. The slaughter performance for these two groups was summarized in Table 3. The percentages of breast muscle yield were 9.85 % for the LPB group and 14.15 % for the HPB group, respectively. No significant differences (*P* > 0.05) were observed between groups in live weight before slaughter, and percentages of dressing, half-eviscerated yield, eviscerated yield, leg muscle yield, and abdominal fat percentage yield.

**Table 3:** Comparison of slaughter performance between langshan chickens with different percentage of breast muscle yield.

Items	Treatment		*P* value	CV (LPB)	CV (HPB)
	LPB	HPB			
Live weight before slaughter (kg)	1.89 ± 0.046	1.85 ± 0.045	0.637	6.47 %	6.51 %
Dressing percentage (%)	86.87 ± 0.65	87.31 ± 0.53	0.616	1.97 %	1.62 %
Percentage of half-eviscerated yield (%)	78.85 ± 0.67	77.38 ± 2.00	0.506	2.25 %	6.80 %
Percentage of eviscerated yield (%)	73.68 ± 0.70	72.30 ± 2.00	0.532	2.50 %	7.27 %
Percentage of breast muscle yield (%)	9.85 ± 0.21	14.15 ± 0.15**	0.001	5.70 %	2.74 %
Percentage of leg muscle yield (%)	17.52 ± 0.37	18.78 ± 0.78	0.172	5.64 %	10.97 %
Percentage of abdominal fat percentage yield (%)	2.09 ± 0.23	1.43 ± 0.31	0.115	9.80 %	14.64 %

Note: Data are expressed as the mean ± SEM, *n* = 7, *P < 0.05, ** *P* < 0.01, *** *P* < 0.001. LPB, low percentage of breast muscle yield group; HPB, high percentage of breast muscle yield group.

### Elevated breast muscle yield correlates with enhanced meat quality in Langshan chickens

Measurements of meat quality indicators were shown in Table 4. Compared to the LPB group, shear force value was significantly lower in the HPB group (*P* = 0.034), indicating improved meat tenderness. For water-holding capacity indicators, the drip loss decreased significantly from 3.07 % in the LPB group to 2.5 % in the HPB group (*P* = 0.012), however, no significant difference (*P* > 0.05) was found in cooking loss. In addition, no differences were found in pH value measured 45 min and 24 h post-slaughter, or the L* (lightness), a* (redness), and b* (yellowness) values for meat color. The above results suggest that meat quality of Langshan chickens in the HPB group was superior in tenderness and water-holding capacity to that of the LPB group.

**Table 4:** Comparison of meat quality between Langshan chickens with different percentage of breast muscle yield.

Items	Treatment		*P* value	CV (LPB)	CV (HPB)
	LPB	HPB			
pH_45min_	6.39 ± 0.14	6.36 ± 0.14	0.634	5.60 %	5.90 %
pH_24h_	5.81 ± 0.044	5.91 ± 0.060	0.187	2.00 %	2.70 %
L*	48.63 ± 0.75	48.78 ± 0.60	0.878	4.09 %	3.23 %
a*	3.12 ± 0.33	3.21 ± 0.31	0.847	2.11 %	5.83 %
b*	2.98 ± 0.51	3.17 ± 0.35	0.765	9.00 %	13.24 %
Drip loss (%)	3.07 ± 0.12	2.50 ± 0.11*	0.012	2.45 %	2.31 %
Cooking loss (%)	7.83 ± 0.79	8.37 ± 0.64	0.634	1.13 %	3.57 %

Note: Data are expressed as the mean ± SEM, *n* = 7, *P < 0.05, ** *P* < 0.01, *** *P* < 0.001. LPB, low percentage of breast muscle yield group; HPB, high percentage of breast muscle yield group.

### Decreased diameter and increased density of muscle fibers contribute to elevated breast muscle yield in Langshan chickens

**Fig. 1:**
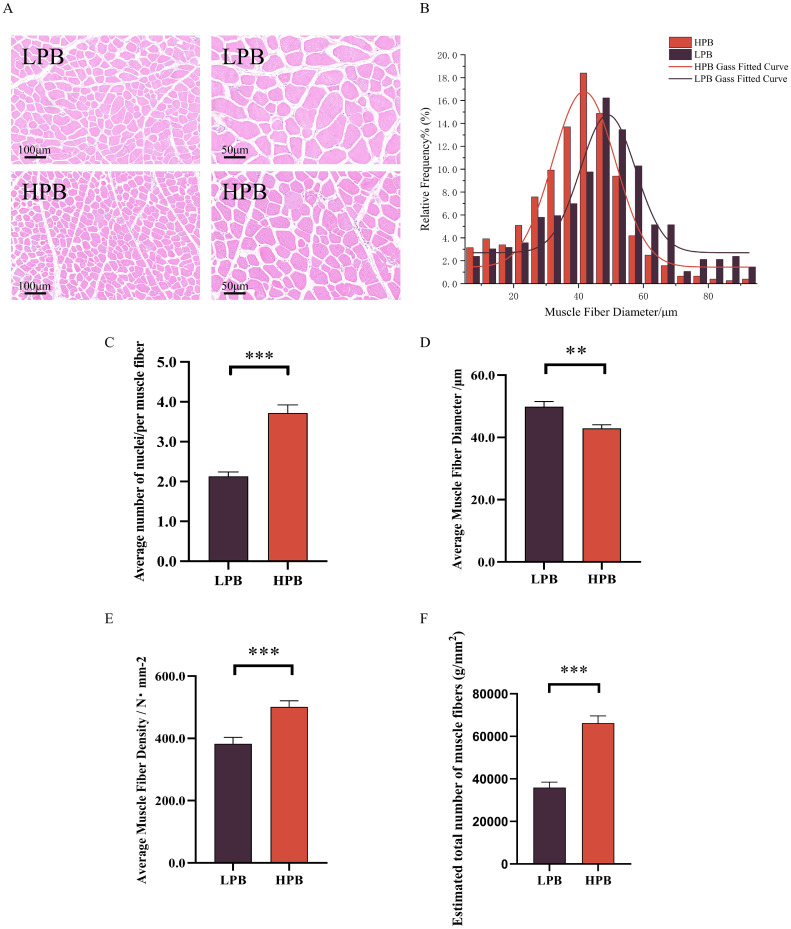
Histological analysis of muscle fiber characteristics in Langshan chickens with different percentage of breast muscle yield. (A) Representative images of HE-staining of breast muscle (scale bar = 100 or 50 μm). (B) Distribution diagrams of muscle fiber diameters. (C) Average muscle fiber diameter. (D) Average muscle fiber density. (E) Average number of nuclei per muscle fiber.LPB. (F) Estimated total muscle fiber numbers in the pectoral muscle. Muscle fiber number = Left pectoral muscle weight / Average muscle fiber cross-sectional area (with exact muscle weight measured to 0.01 g). LPB: Low percentage of breast muscle yield group; HPB: High percentage of breast muscle yield group. Data were expressed as the mean ± SEM, *n* = 7. *P < 0.05, ** *P* < 0.01, *** *P* < 0.001.

The histological analysis of muscle fibers in two groups of Langshan chickens was shown in Fig. 1A. Analysis of the diameter distribution showed that muscle fibers in the HPB group were primarily 30–50 μm in diameter, whereas those in the LPB group were mostly 40–60 μm (Fig. 1B). The average muscle fiber diameter (Fig. 1C) of the HPB group was significantly smaller than those of the LPB group (*P* < 0.01), while the average muscle fiber density (Fig. 1D) of the HPB group was significantly higher (*P* < 0.001). In addition, the average number of nuclei per muscle fiber was higher in the HPB group than the LPB group (Fig. 1E). Combined with the fact that breast muscle weight was higher in the HPB group, the above results suggest that the number of muscle fibers was greater in the breast muscle of this group. Then, we estimated the number of muscle fibers by calculating “Left pectoral muscle weight / Average muscle fiber cross-sectional area (with exact muscle weight measured to 0.01 g) ". The results confirmed that the HPB group possessed a significantly higher total muscle fiber number than the LPB group (Fig. 1F). Therefore, these results suggest that the elevated breast muscle yield in the HPB group might be attributable to a greater number of muscle fibers, rather than larger muscle fiber size.

### Enhanced myogenic capacity leads to elevated breast muscle yield

**Fig. 2:**
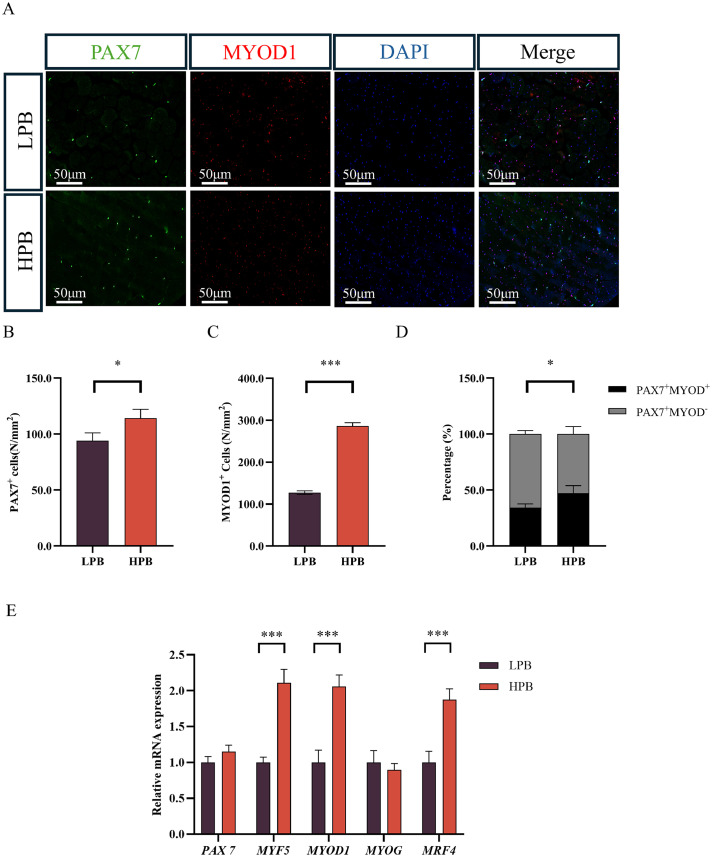
Comparation of myogenic capacity differences in Langshan chickens with different percentage of breast muscle yield. (A) Representative images of PAX7 and MYOD1 co-localization (scale bar = 50 μm). (B) Number of PAX7^+^ satellite cells (SCs) per unit area. (C) Number of MYOD1^+^ SCs per unit area. (D) Percentage of PAX7^+^/ MYOD1^+^ and PAX7^+^/MYOD1^+^ cells among Pax7+ SCs. (E) Relative mRNA expression of myogenic regulatory factors. LPB: Low percentage of breast muscle yield group; HPB: High percentage of breast muscle yield group. Data were expressed as the mean ± SEM, *n* = 7. *P < 0.05, ** *P* < 0.01, *** *P* < 0.001.

The myogenic capacity of the two groups was assessed by analyzing satellite cell (SC) activity and the expression of myogenic regulatory genes (Fig. 2A-E). Immunofluorescence staining of PAX7 and MYOD1 identified SCs in different cellular states (Fig. 2A). Statistical analysis showed that the number of both PAX7^+^ (*P* < 0.05) and MYOD1^+^ (*P* < 0.001) SCs per unit area were significantly higher in the HPB group than in the LPB group (Fig. 2B and C). Furthermore, the percentage of PAX7^+^/MYOD1^+^ SCs was significantly increased in the HPB group, while the percentage of PAX7^+^/MYOD1^-^ SCs decreased (Fig. 2D). In addition, the relative expression levels of *MYF5, MYOD1*, and *MRF4* were higher in the HPB group (Fig. 2E), while no differences were found for *PAX7* and *MYOG* (*P* < 0.001). Collectively, the above findings indicate that the proliferation and myogenic differentiation of SCs were enhanced in the HPB group.

### Increased protein deposition capacity in Langshan chickens with elevated breast muscle yield

**Fig. 3:**
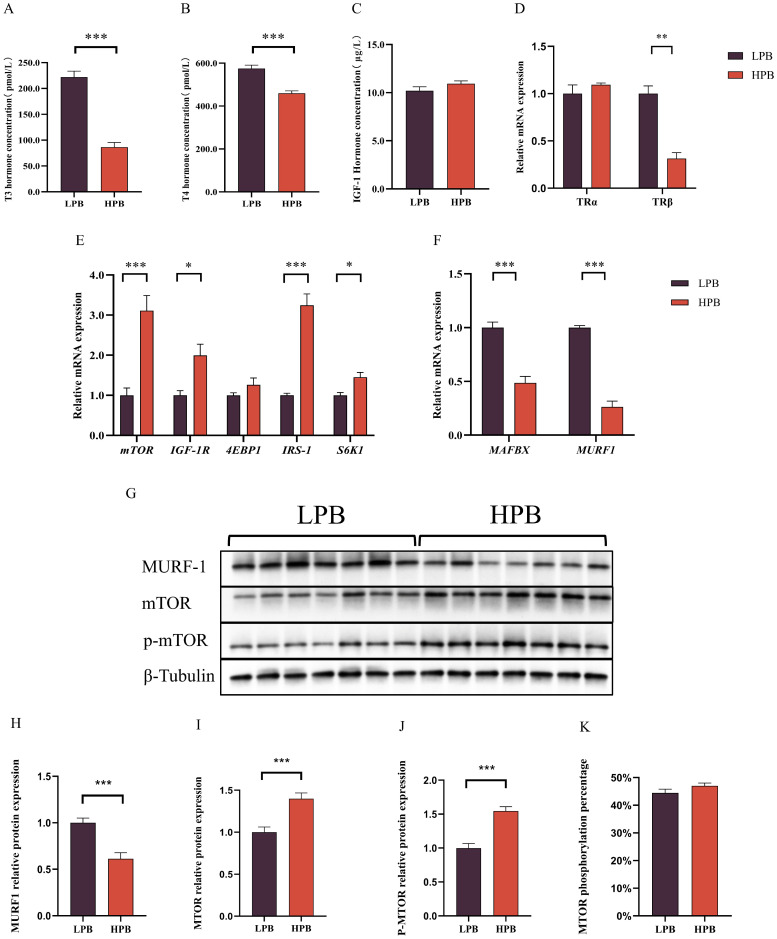
Determination of serum hormone contents and relative mRNA expression levels of genes related to protein synthesis and degradation in Langshan chickens with different percentage of breast muscle yield. (A-C) Concentrations of triiodothyronine (T3), thyroxine (T4), and insulin-like growth factor-1 (IGF-1). (D-E) Relative mRNA expression of genes related to protein synthesis and degradation. (F) Relative mRNA expression of thyroid hormone receptors *TRα* and *TRβ* in the pectoral muscle. (G) Representative Western blot images of MURF-1, mTOR, and phosphorylated mTOR (p-mTOR). (H–J) Quantification of MURF-1, mTOR, and p-mTOR protein expression levels. (K) Phosphorylation ratio of mTOR. LPB: Low percentage of breast muscle yield group; HPB: High percentage of breast muscle yield group. Data were expressed as the mean ± SEM, *n* = 7. *P < 0.05, ** *P* < 0.01, *** *P* < 0.001.

Thyroid hormones regulate the balance between protein synthesis and degradation. Compared with the LPB group, serum concentrations of T3 and T4 in the HPB group were significantly decreased (*P* < 0.001), while no significant differences were found for IGF-1 (*P* > 0.05, Fig. 3A-C). Expression levels of genes related to protein synthesis and degradation were provided in Fig. 3D and E. The HPB group exhibited significant higher expression levels of protein synthesis-related genes (*mTOR, IGF-1R, IRS-1*, and *S6K1*) and significant lower expression levels of protein degradation-related genes (*MAFBX* and *MURF1*). Therefore, the combined effect of increased protein synthesis and decreased degradation promoted breast muscle growth in the HPB group. To further explore the potential involvement of thyroid hormone signaling in breast muscle development, the mRNA expression levels of thyroid hormone receptors *TRα* and *TRβ* were examined in the pectoral muscle of HPB and LPB chickens. As shown in Fig. 3F, no significant difference was observed in *TRα* expression between the two groups, whereas *TRβ* expression was significantly lower in the HPB group compared with the LPB group (*P* < 0.01). In addition, key proteins involved in muscle protein metabolism were analyzed by Western blotting (Fig. 3G). Compared with the LPB group, the HPB group exhibited significantly lower expression of the protein degradation–related marker MURF-1 (Fig. 3H, *P*< 0.001), while the expression levels of mTOR and phosphorylated mTOR (p-mTOR) were markedly increased (Fig. 3I and J, *P* < 0.001). However, the phosphorylation ratio of mTOR did not differ significantly between the two groups (Fig. 3K).

### Untargeted metabolomics analysis

**Fig. 4:**
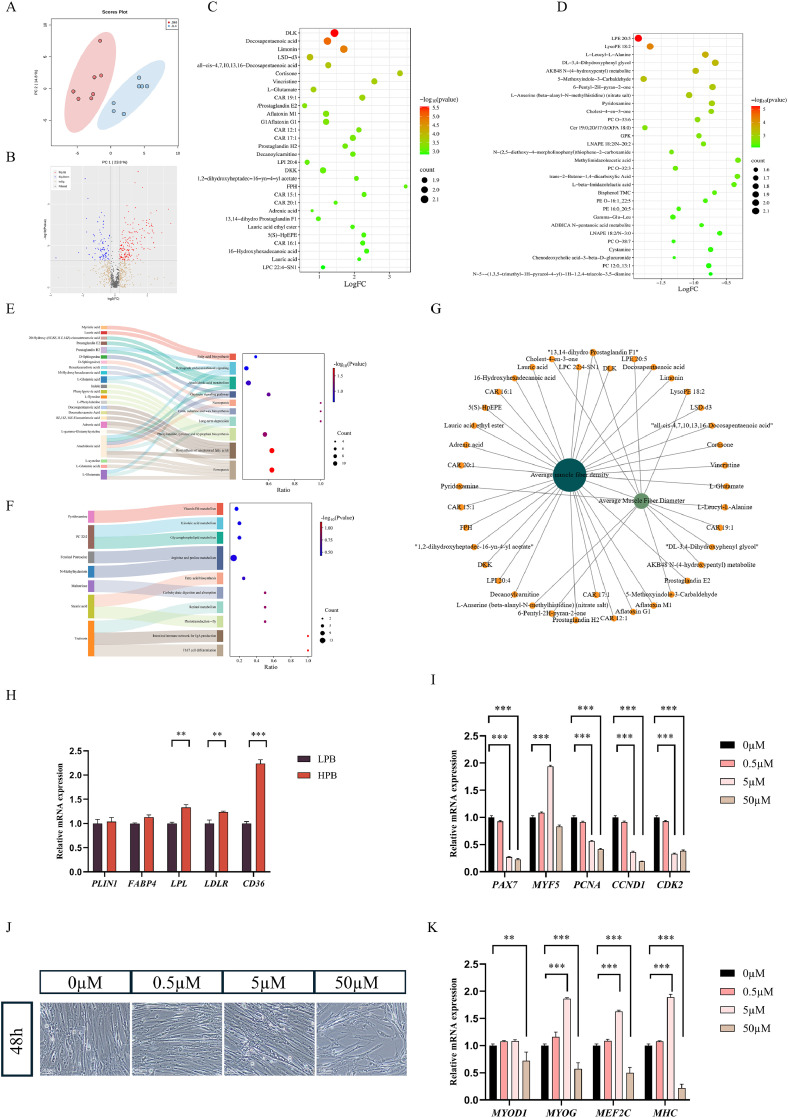
Untargeted metabolomic analysis of breast muscle in Langshan chickens with different percentage of breast muscle yield. (A) Principal component analysis of samples from HPB and LPB groups. (B) Volcano plot showing differential metabolites. (C-D) Bubble plot showing the top 30 up-regulated or down-regulated differential metabolites in the HPB group. (E) Mulberry plot showing top 10 KEGG pathway enriched for up-regulated or down-regulated differential metabolites. (G) Correlation analysis of muscle fiber characteristics, and differential metabolites. (H) Relative mRNA expression of lipid metabolism–related genes. (I) Relative mRNA expression of proliferation-related genes (*PAX7, MYF5, PCNA, CCND1,* and *CDK2*) after 24 h treatment of different concentrations of DPA in growth medium. (J) Representative morphological images of myotube formation following 48 h of differentiation with different concentrations of DPA treatment. (K) Relative mRNA expression of myogenic differentiation–related genes (*MYOD1, MYOG, MEF2C,* and *MHC*) after 48 h of differentiation. LPB: Low percentage of breast muscle yield group; HPB: High percentage of breast muscle yield group. Data were expressed as the mean ± SEM, *n* = 7. *P < 0.05, ** *P* < 0.01, *** *P* < 0.001.

The PCA plot of untargeted metabolomics showed obvious separation between groups, indicating significant differences between groups (Fig. 4A). The screening and visualization of differential metabolites are shown in Fig. 4B-D. Differential metabolites were screened based on the VIP value of the OPLS-DA model, combined with the *P*-values. The screening criteria for differential metabolites were set at |log_2_FC| ≥ 1, OPLS-DA_VIP ≥ 1, and *P*-value ≤ 0.05. A total of 253 differential metabolites were identified, including 169 upregulated metabolites and 84 downregulated metabolites. In addition, the relevant information of the top 30 upregulated and top 30 downregulated differential metabolites in the sequencing was presented (Fig. 4C-D).

The KEGG metabolic pathway enrichment analysis was conducted based on up-or down-regulated differential metabolites in the HPB group compared to those of the LPB group. As shown in Fig. 4E, the upregulated metabolic pathways were mainly enriched in ferroptosis, biosynthesis of unsaturated fatty acids, biosynthesis of phenylalanine, tyrosine and tryptophan, arachidonic acid metabolism, and fatty acid biosynthesis. The downregulated metabolic pathways (Fig. 4F) were mainly associated with immune responses (Th17 cell differentiation, intestinal immune network for IgA production), carbohydrate digestion and absorption, lipid synthesis and metabolism (fatty acid biosynthesis, glycerophospholipid metabolism, linoleic acid metabolism), amino acid metabolism (arginine and proline metabolism), and vitamin metabolism (vitamin B6 metabolism, retinol metabolism).

The correlation analysis between two key muscle fiber characteristics and differential metabolites were shown in Fig. 4G. Average muscle fiber density was widely associated with various metabolites, including lipid-related compounds (e.g., carnitine derivatives such as CAR 16:1, CAR 20:1, and CAR 15:1; phospholipid derivatives such as LPC 22:4 - SN1 and LPE 20:5), amino acid-related metabolites (e.g., pyridoxamine and L-anserine), and oxidized lipids (e.g., 13,14-dihydroprostaglandin F1 and prostaglandin H2). Average muscle fiber diameter was also associated with several metabolites, including carnitines (CAR 19:1 and CAR 17:1), amino acid derivatives (L-leucyl-L-alanine and L-glutamic acid), and other compounds (e.g., DL-3,4-dihydroxyphenylethylene glycol and prostaglandin E2).

In addition, lipid metabolism–related genes were examined by RT-qPCR. As shown in Fig. 4H, the expressions of *PLIN1* and *FABP4* did not differ significantly between the two groups. In contrast, the HPB group exhibited significantly higher expressions of *LPL, LDLR*, and *CD36* compared with the LPB group (*P* < 0.01 or *P* < 0.001).

Based on the above integrative analyses, docosapentaenoic acid (DPA) was identified as one of the most differential expressed metabolites. We conducted in vitro cell culture experiments to investigate the effects of DPA at different concentrations (0, 0.5, 5, and 50 μM) on the proliferation and differentiation of SCs (Fig. 4I-K). After culturing for 24 h in growth medium, the expression of proliferation-related genes, including *PAX7, PCNA, CCND1*, and *CDK2*, was significantly decreased following 5 and 50 μM DPA treatment (Fig. 4I), indicating an inhibitory effect of DPA on proliferative activity. After culturing for 48 h in the differentiation induction medium, morphological observations showed that treatment with 5 μM DPA markedly enhanced myotube formation, whereas 50 μM DPA impaired myotube formation compared with the control group (Fig. 4J). Consistently, the expression of myogenic differentiation–related genes, including *MYOG, MEF2C*, and *MHC*, was significantly up-regulated in the 5 μM DPA–treated group, while 50 μM DPA significantly reduced their expression (Fig. 4K). These results demonstrate that DPA exerts concentration-dependent effects on the differentiation of SCs.

## Discussion

Genetic selection and advances in nutrition have dramatically enhanced the growth rate and breast muscle yield of modern commercial broilers. This focus on productivity, however, has often resulted in reduced intramuscular fat deposition and compromised meat quality (Hocquette et al., 2010). In Asian markets, consumers prefer local chicken breeds for their superior meat quality, but these breeds typically exhibited slower growth rate and lower breast muscle yield (Deng et al., 2022). Thus, a major industry goal is the simultaneous enhancement of both muscle growth and meat quality in local chickens; however, effective methods to achieve this remain elusive. This study utilized two groups of Langshan chickens with high or low percentage of breast muscle yield from a total of 70 chickens with comparable body weight. Notably, the HPB group demonstrated both enhanced myogenic potential and improved meat quality compared to the LPB group. To elucidate the regulatory mechanisms underlying this simultaneous improvement, we compared the characteristics of muscle fibers, myogenic activities of satellite cells (SCs), protein deposition capacity, and metabolic profiles between the two groups.

Our results showed that elevated breast muscle yield does not necessarily impair meat quality, instead, the drip loss and shear force were significantly reduced in the HPB group. Drip loss reflects the water-holding capacity of muscle, whereas shear force is directly related to tenderness; both are essential indicators for evaluating meat quality from the consumer perspective. Shear force shows a positive correlation with diameter and a negative correlation with the density of muscle fiber, while drip loss displays a similar correlation pattern (Honikel et al., 1998; Baéza et al., 2022). Consistently, we found that muscle fibers in the HPB group were smaller in diameter but denser. This, combined with the higher breast muscle weight, indicates a greater number of muscle fibers in the HPB group. While changes in fiber diameter and density are often used as indirect indicators of muscle fiber hyperplasia, we further sought to provide more quantitative support for differences in muscle fiber number between groups. Therefore, total muscle fiber number was estimated based on left pectoral muscle weight and average muscle fiber cross-sectional area. Using this approach, we confirmed that the HPB group exhibited a significantly higher estimated total muscle fiber number compared with the LPB group. Therefore, the simultaneous improvement in muscle growth and meat quality in the HPB group can be attributed to a greater number of muscle fibers (Zhao et al., 2011; Weng et al., 2022). This mechanism represents a promising strategy for future high-quality chicken breeding.

The myogenic capacity of SCs determines the growth rate of breast muscles (Collins et al., 2005; Valdez et al., 2000; Hernández-Hernández et al., 2017). Yin et al. (2013) reported that SCs promote muscle growth by activating, proliferating, and differentiating into myoblasts, which subsequently fuse with existing muscle fibers or form new ones. Consistently, the HPB group exhibited significantly greater number of SCs in the state of both proliferation (PAX7^+^ or PAX7^+^/MYOD1^-^) and differentiation (MYOD1^+^ or PAX7^+^/MYOD1^+^). Thus, the enhanced SCs activity may serve as the key driving force for breast muscle growth in the HPB group.

In addition to the myogenic activity of SCs, the hypertrophy of breast muscle also depends heavily on the protein deposition capacity of mature muscle fibers (Choi et al., 2014; Chen et al., 2024). Net protein deposition occurs when protein synthesis exceeds protein degradation. The mTOR signaling pathway plays a major role in promoting protein synthesis in response to growth signals (Laplante et al., 2012). IRS-1, a major adaptor in insulin signaling, activates the downstream PI3K–AKT–mTOR cascade, thereby enhancing protein synthesis (White, 2002). IGF-1R, the receptor for insulin-like growth factor-1 (IGF-1), mediates activation of the PI3K–AKT–mTOR pathway, facilitating muscle growth and protein synthesis (Le Roith et al., 2001). S6K1, a downstream effector of mTOR, phosphorylates ribosomal protein S6, further stimulating protein synthesis (Magnuson et al., 2012; Yang et al., 2022). Upregulation of these genes suggests that Langshan chickens in the HPB group may enhance protein synthesis via activation of the insulin/IGF-1–mTOR pathway. In addition, an increased number of nuclei in muscle fibers were observed in the HPB group. This finding also reflected an enhanced protein synthesis capacity, as nuclei newly incorporated from SCs exhibit high transcriptional activity (Gussoni et al., 1999). Regarding protein degradation, muscle-specific ubiquitin ligases such as MAFBX and MURF1 play key roles (Bodine et al., 2001; Gomes et al., 2001). The significantly reduced expression of MAFBX and MURF1 in the HPB group indicates suppression of protein degradation pathways.

Interestingly, despite the enhanced protein deposition capacity, serum T3 and T4 concentrations were lower in the HPB group than in the LPB group. Thyroid hormones are generally known to stimulate muscle protein turnover, and both stimulatory and inhibitory effects on muscle growth have been reported depending on developmental stage, hormone concentration and receptor context (Dentice et al., 2014). One possible explanation is that a lower systemic thyroid hormone level combined with locally regulated thyroid hormone signaling in muscle may reduce energy-demanding protein turnover while maintaining effective protein synthesis via the insulin/IGF-1–mTOR pathway, thereby improving metabolic efficiency in HPB chickens. Nevertheless, the precise interaction between thyroid hormones and mTOR-mediated protein synthesis in Langshan chickens requires further investigation(De Stefano et al., 2021;Ucci et al., 2019).

The changes of myogenic capacity, protein deposition capacity, and meat quality are closely related to changes in metabolic profiles. Our untargeted metabolomic analysis revealed distinct metabolic profiles between the HPB and LPB groups, with unsaturated fatty acid biosynthesis significantly upregulated in the HPB group. Unsaturated fatty acids perform multiple essential functions in muscle growth and maintenance, serving as major components of cellular membranes and modulating membrane fluidity and function (Hagve, 1988). Moreover, some unsaturated fatty acids act as signaling molecules that regulate intracellular processes of myogenesis such as proliferation, differentiation, and gene expression (Jump et al., 2002). Xu et al. (2018) demonstrated that docosahexaenoic acid (DHA) enhances SCS differentiation in bovine muscle by promoting myotube fusion and increasing the expression of *MYOG* and *MYH3*. Similarly, Shin et al. (2017) reported that DHA alleviated dexamethasone-induced muscle atrophy in C2C12 myoblasts by restoring *MYOD1* expression and enhancing myogenesis. Therefore, the upregulation of unsaturated fatty acid biosynthesis in the HPB group observed here may promote SCs differentiation, resulting in higher muscle fiber density and smaller fiber diameter. Correlation network analysis between muscle fiber characteristics and differential metabolites indicated that muscle fiber characteristics are influenced by interconnected processes, including lipid metabolism (e.g., carnitine-mediated fatty acid oxidation and phospholipid remodeling), amino acid metabolism, and oxidized lipid–driven signaling pathways. Future studies should focus on the role of identified metabolites and associated metabolic pathways, such as those involving unsaturated fatty acids, in promoting muscle growth.

In the present study, untargeted metabolomics revealed that several metabolites involved in unsaturated fatty acid biosynthesis were differentially abundant between the LPB and HPB groups. Although the current analysis is correlative, these changes are biologically consistent with the observed differences in breast muscle yield and meat quality. Unsaturated fatty acids are key components of membrane phospholipids and modulate membrane fluidity and stability; increased levels of specific long-chain unsaturated fatty acids in the HPB group may help maintain the integrity of muscle cell membranes during postmortem storage, thereby reducing drip loss and improving water-holding capacity. In addition, unsaturated fatty acids can act as signaling molecules that activate lipid-sensitive transcription factors (e.g., PPARs) and intersect with the IGF-1–mTOR axis, potentially supporting a metabolic milieu that favors protein deposition in pectoral muscle. Consistent with this notion, several unsaturated fatty acid–related metabolites showed positive correlations with breast muscle yield and negative correlations with shear force and drip loss in our correlation network analysis. Taken together, these findings suggest that enhanced unsaturated fatty acid metabolism may represent an important metabolic signature linking increased breast muscle yield to improved meat quality in Langshan chickens, although targeted quantification and functional validation are still required. Our RT-qPCR results showed the upregulation of *LPL, LDLR*, and *CD36* expression in the HPB group, which indicates altered lipid handling capacity in the pectoral muscle, potentially facilitating fatty acid uptake and utilization. Such metabolic adaptations may contribute to membrane lipid remodeling and energy supply during muscle fiber development. These findings further support the notion that improved muscle growth in HPB chickens is linked to metabolic efficiency rather than increased fat accumulation (Goldberg et al., 2009;Son et al., 2018; Ramos-Jiménez et al., 2022).

Fatty acids have been reported to exert concentration-dependent effects on myogenic differentiation (Bhullar and Templeman, 2016; Isesele et al., 2021; Risha et al., 2021). In the present study, the effects of DPA on satellite cell proliferation and differentiation were examined. DPA treatment in growth medium significantly reduced the expression of proliferation-related genes. In contrast, when SCs were subjected to a differentiation protocol, moderate DPA supplementation (5 μM) markedly promoted myotube formation and enhanced the expression of myogenic differentiation markers, whereas excessive DPA exposure impaired differentiation. These findings indicate that DPA may act as a metabolic modulator influencing satellite cell fate decisions in a concentration-dependent manner. Specifically, appropriate DPA levels may facilitate the transition toward myogenic differentiation, whereas excessive exposure may disrupt cellular homeostasis. Together, these results provide functional evidence linking differential metabolites identified by metabolomic analysis to satellite cell behavior and skeletal muscle development.

From an applied perspective, the cellular and molecular indicators identified here (e.g., higher satellite cell activity, greater muscle fiber density, and specific unsaturated fatty acid–related metabolites) may serve as potential markers for breeding programs aimed at selecting Langshan chickens with both high breast muscle yield and desirable meat quality. In addition, nutritional strategies that modulate dietary lipid sources and promote beneficial unsaturated fatty acid profiles could be explored to further support this phenotype.

Limitation: Though all birds in this study were obtained from a single Langshan population maintained at one breeding farm and belonged to the same generation, we cannot completely exclude the potential influence of underlying genetic heterogeneity on the observed phenotypic differences. Future studies combining controlled breeding designs and genomic information will be necessary to confirm whether the identified cellular and metabolic features can be used as robust selection criteria across generations and lines. We explicitly acknowledge that our findings are limited to males of one age and may not directly generalize to females or chickens at other ages. Due to the relatively smaller number of replicates used in this study (*n* = 7), future studies should validate our findings in larger populations. In addition, more molecular methods related to the evaluation of SCs function especially at early ages and their contributions to breast muscle yields should be conducted to better explain the underlying mechanisms.

In conclusion, our study provides a novel insight that an elevated percentage of breast muscle yield does not impair meat quality in Langshan chickens but instead improves tenderness and water-holding capacity. The reasons underlying these improvements may stem from the decreased diameter and increased density of muscle fibers in the HPB group. Therefore, increasing the number of myofibers represents a promising strategy for the simultaneous enhancement of breast muscle yield and meat quality. Furthermore, we identified multiple metabolites with the potential to promote breast muscle growth, particularly unsaturated fatty acids. Collectively, our findings provide a valuable theoretical basis for simultaneously increasing breast muscle yield and meat quality in Langshan chickens.

## CRediT authorship contribution statement

**Junjie Chen:** Writing – review & editing, Writing – original draft, Visualization, Validation, Methodology, Formal analysis, Data curation, Conceptualization. **Xiuze Zhang:** Writing – review & editing, Methodology. **Lin Zhang:** Supervision, Resources. **Tong Xing:** Supervision, Resources. **Feng Gao:** Supervision, Resources, Funding acquisition. **Liang Zhao:** Writing – review & editing, Visualization, Supervision, Resources, Project administration, Methodology, Investigation, Funding acquisition, Conceptualization.

## Disclosures

The authors declare that they have no known competing financial interests or personal relationships that could have appeared to influence the work reported in this paper.

## Appendix Supplementary materials

Image, application 1
