# Supplementary material for: Simultaneous improvement of breast muscle yield and meat quality in Langshan chickens
Source: Poult Sci. 2026 Jan 30;105(4):106552. doi: 10.1016/j.psj.2026.106552 (PMC12919249; doi:10.1016/j.psj.2026.106552)
Supplement: Supplementary file 1 [file mmc1.docx]

**TableS1.** Relevant Data of the Top 30 Upregulated Differential Metabolites in Langshan Chickens with Different Breast Muscle Yields.

| Serial No | Name | Molecular Weight | RT [min] | m/z | VIP | JH.aver | JL.aver | P-value |
| --- | --- | --- | --- | --- | --- | --- | --- | --- |
| 1 | DLK | 374.21991 | 10.129 | 375.22719 | 2.145599333 | 172222276.4 | 63393545.04 | 0.00000277 |
| 2 | \| Docosapentaenoic acid \| \| --- \| | 330.25579 | 10.121 | 329.24852 | 2.087012446 | 261673964.6 | 110381751.2 | 0.00000953 |
| 3 | Limonin | 487.22765 | 4.882 | 488.23493 | 2.082253302 | 14884120.8 | 4569815.06 | 0.00001623 |
| 4 | LSD-d3 | 326.21984 | 10.589 | 327.22712 | 1.968508783 | 1054524648 | 628009642.2 | 0.00012237 |
| 5 | all-cis-4,7,10,13,16-Docosapentaenoic acid | 330.25598 | 10.308 | 353.24523 | 1.947233675 | 199489012.2 | 82915245 | 0.00014962 |
| 6 | Cortisone | 1440.76504 | 5.694 | 721.3898 | 1.922534954 | 57203405.34 | 5782782.41 | 0.0001759 |
| 7 | Vincristine | 1648.81645 | 5.747 | 825.4155 | 1.93433453 | 7681244.96 | 1292256.42 | 0.00020926 |
| 8 | L-Glutamate | 147.05322 | 1.589 | 148.06045 | 1.930707753 | 1254330824 | 698605748.6 | 0.00023511 |
| 9 | CAR 19:1 | 439.36601 | 8.77 | 440.37328 | 1.94078354 | 20043627.52 | 4249542.62 | 0.00027089 |
| 10 | Prostaglandin E2 | 352.2267 | 10.322 | 353.23398 | 1.859280843 | 10709718.82 | 7078743.96 | 0.00046259 |
| 11 | Aflatoxin M1 | 350.03378 | 4.873 | 351.04106 | 1.942416926 | 35578320.95 | 15434998.89 | 0.00051796 |
| 12 | Aflatoxin G1 | 164.02595 | 4.886 | 351.04108 | 1.941367588 | 35588938 | 15465413.77 | 0.00052427 |
| 13 | CAR 12:1 | 359.26704 | 6.591 | 360.2743 | 1.873243979 | 62281193.93 | 14190599.38 | 0.00054787 |
| 14 | CAR 17:1 | 411.33494 | 8.088 | 412.34221 | 1.934563714 | 36051699.99 | 9232813.25 | 0.00060565 |
| 15 | Prostaglandin H2 | 334.21395 | 7.739 | 333.20673 | 1.893158868 | 2166570.93 | 647696.76 | 0.00064474 |
| 16 | Decanoylcarnitine | 315.24089 | 6.426 | 316.24816 | 1.885147871 | 542891732 | 138675023.5 | 0.00075024 |
| 17 | LPI 20:4 | 620.29681 | 8.739 | 619.28954 | 1.872775143 | 815831926.8 | 513446324.6 | 0.00077991 |
| 18 | DKK | 411.21162 | 5.431 | 412.2189 | 1.940706001 | 23845775.94 | 11021570.87 | 0.0007989 |
| 19 | 1,2-dihydroxyheptadec-16-yn-4-yl acetate | 343.27224 | 6.951 | 344.27952 | 1.855869269 | 553599063.5 | 132730788.6 | 0.00082978 |
| 20 | FPH | 762.36252 | 5.582 | 382.18854 | 1.819842814 | 24935359.08 | 2262912.79 | 0.00084325 |
| 21 | CAR 15:1 | 383.30347 | 7.502 | 384.31076 | 1.866905114 | 32497679.53 | 6655289.95 | 0.00090323 |
| 22 | CAR 20:1 | 453.38185 | 9.087 | 454.38913 | 1.82768078 | 1353892713 | 485878539.7 | 0.00095732 |
| 23 | Adrenic acid | 332.27147 | 10.609 | 331.26421 | 1.811786491 | 394620903.6 | 226517490 | 0.00095811 |
| 24 | 13,14-dihydro Prostaglandin F1 | 394.24786 | 10.175 | 393.24058 | 1.849889115 | 36124296.14 | 18188696.53 | 0.00099075 |
| 25 | Lauric acid ethyl ester | 228.20824 | 7.561 | 227.20096 | 1.833107695 | 21680500.27 | 5582155.22 | 0.0010573 |
| 26 | 5(S)-HpEPE | 167.10562 | 8.107 | 333.20413 | 1.859430146 | 5734238.23 | 1183159.43 | 0.0010748 |
| 27 | CAR 16:1 | 397.3193 | 7.763 | 398.32659 | 1.859333264 | 2066758602 | 436276222 | 0.0011333 |
| 28 | 16-Hydroxyhexadecanoic acid | 254.22413 | 7.758 | 253.21686 | 1.85410816 | 44457327.34 | 8714347.45 | 0.0011696 |
| 29 | Lauric acid | 200.1768 | 6.941 | 199.16952 | 1.816458796 | 7996218.15 | 1802734 | 0.0012279 |
| 30 | LPC 22:4-SN1 | 571.36377 | 9.682 | 572.37097 | 1.82909999 | 15155066.45 | 6988177.83 | 0.0014614 |

**TableS2.** Relevant Data of the Top 30 Downregulated Differential Metabolites in Langshan Chickens with Different Breast Muscle Yields.

| Serial No | Name | Molecular Weight | RT [min] | m/z | VIP | JH.aver | JL.aver | P-value |
| --- | --- | --- | --- | --- | --- | --- | --- | --- |
| 1 | LPE 20:5 | 499.26908 | 7.731 | 500.27637 | 2.111085584 | 2095879.37 | 7605900.15 | 0.00000600 |
| 2 | LysoPE 18:2 | 477.28629 | 7.732 | 500.27551 | 2.011339615 | 2356841.76 | 7528882.67 | 0.00005375 |
| 3 | L-Leucyl-L-Alanine | 202.13181 | 4.542 | 203.13909 | 1.95613412 | 162422562 | 287205435.2 | 0.0002569 |
| 4 | DL-3,4-Dihydroxyphenyl glycol | 170.05803 | 4.494 | 171.06531 | 1.983478953 | 3611363.11 | 5762966.64 | 0.00036448 |
| 5 | AKB48 N-(4-hydroxypentyl) metabolite | 762.4752 | 5.379 | 382.24487 | 1.890706751 | 14867123.26 | 29276316.85 | 0.00042151 |
| 6 | 5-Methoxyindole-3-Carbaldehyde | 175.06343 | 5.792 | 176.07075 | 1.868664757 | 2297901.05 | 7881004.21 | 0.0004826 |
| 7 | 6-Pentyl-2H-pyran-2-one | 166.09982 | 0.672 | 167.1071 | 1.917983546 | 10220973.88 | 16755351.37 | 0.00067555 |
| 8 | L-Anserine (beta-alanyl-N-methylhistidine) (nitrate salt) | 240.12249 | 0.572 | 121.06864 | 1.89426903 | 181913025.5 | 383053782 | 0.00070879 |
| 9 | Pyridoxamine | 168.09006 | 4.961 | 169.09727 | 1.85725076 | 34154143.73 | 56335148.07 | 0.00091844 |
| 10 | Cholest-4-en-3-one | 384.33874 | 0.161 | 385.34601 | 1.728789957 | 25262355.51 | 42061095.51 | 0.0014025 |
| 11 | PC O-33:6 | 721.50578 | 11.224 | 722.51267 | 1.752806584 | 34331449.16 | 65935013.07 | 0.0017472 |
| 12 | Cer 19:0;2O/17:0;O(FA 18:0) | 849.81556 | 11.273 | 848.80829 | 1.691488512 | 7660175.85 | 25858198.34 | 0.0021927 |
| 13 | GPK | 644.32707 | 4.986 | 323.17081 | 1.725023758 | 3453825.52 | 6559191.38 | 0.0023014 |
| 14 | LNAPE 18:2/N-20:2 | 767.54744 | 11.034 | 766.54013 | 1.654798484 | 3596462.47 | 6515562.56 | 0.002833 |
| 15 | N-(2,5-diethoxy-4-morpholinophenyl)thiophene-2-carboxamide | 752.29296 | 5.804 | 377.15376 | 1.63127856 | 1306529.41 | 3322533.89 | 0.0028524 |
| 16 | Methylimidazoleacetic acid | 140.05855 | 0.147 | 141.06583 | 1.757994478 | 84016261.43 | 105038742.7 | 0.0031597 |
| 17 | PC O-32:3 | 713.53553 | 11.211 | 714.54255 | 1.68145377 | 9570937.51 | 23272315.37 | 0.0033371 |
| 18 | trans-2-Butene-1,4-dicarboxylic Acid | 144.04236 | 1.741 | 145.04958 | 1.720085775 | 1189805607 | 1497223117 | 0.0033626 |
| 19 | L-beta-Imidazolelactic acid | 156.05351 | 0.291 | 157.06079 | 1.691583713 | 25422105.67 | 33209636.74 | 0.0042981 |
| 20 | Bisphenol TMC | 327.21575 | 5.485 | 328.22302 | 1.62083621 | 82039774.39 | 131928031.9 | 0.0044245 |
| 21 | PE O-16:1_22:5 | 749.53575 | 11.741 | 750.54302 | 1.616965915 | 92883964.96 | 164082520.4 | 0.0053036 |
| 22 | PE 16:0_20:5 | 737.50029 | 9.877 | 736.49302 | 1.604036285 | 4274667.97 | 10093366.98 | 0.0055943 |
| 23 | Gamma-Glu-Leu | 260.1373 | 4.701 | 261.14457 | 1.700857569 | 58046815.67 | 145252590.4 | 0.0058172 |
| 24 | ADBICA N-pentanoic acid metabolite | 373.19543 | 4.852 | 372.18816 | 1.580667567 | 2079135.3 | 3844373.15 | 0.006046 |
| 25 | LNAPE 18:2/N-3:0 | 533.31109 | 9.031 | 532.30381 | 1.673217646 | 5328484.5 | 8090325.18 | 0.0062944 |
| 26 | PC O-38:7 | 789.56769 | 10.182 | 790.57491 | 1.533029103 | 3784794141 | 9392411717 | 0.0065033 |
| 27 | Cystamine | 152.04495 | 5.236 | 153.05222 | 1.671909759 | 31697238.93 | 53077892.69 | 0.0066152 |
| 28 | Chenodeoxycholic acid-3-beta-D-glucuronide | 568.32236 | 4.799 | 285.16846 | 1.501354075 | 1855944.66 | 4577952.92 | 0.0066678 |
| 29 | PC 12:0_13:1 | 633.43656 | 10.152 | 634.44378 | 1.684299725 | 10221871.42 | 17438136.98 | 0.0072399 |
| 30 | N~5~-(1,3,5-trimethyl-1H-pyrazol-4-yl)-1H-1,2,4-triazole-3,5-diamine | 185.14169 | 5.716 | 208.13091 | 1.525750037 | 31876773.31 | 53510955.82 | 0.0078775 |

RT[min], Retention Time [minute];m/z,Mass-to-Charge Ratio;VIP,Variable Importance in Projection;JL.aver,Average Metabolite Concentration in Breast Muscle of Low Breast Muscle Yield Group;JH.aver,Average Metabolite Concentration in Breast Muscle of High Breast Muscle Yield Group; n= 7.
